# Neural Correlate Differences in Number Sense Between Children With Low and Middle/High Socioeconomic Status

**DOI:** 10.3389/fpsyg.2020.534367

**Published:** 2020-10-20

**Authors:** Qing Bao, Li Jin Zhang, Yuan Liang, Yan Bang Zhou, Gui Li Shi

**Affiliations:** ^1^School of Psychology, Shaanxi Normal University, Xi’an, China; ^2^School of Journalism and Communication, Ningxia University, Yinchuan, China; ^3^Shaanxi Provincial Key Research Center of Child Mental and Behavioral Health, Xi’an, China; ^4^Shaanxi Key Laboratory of Behavior and Cognitive Neuroscience, Xi’an, China; ^5^Zhou Enlai School of Government, Nankai University, Tianjin, China; ^6^School of Mechano-Electronic Engineering, Xidian University, Xi’an, China

**Keywords:** low socioeconomic status, event-related potentials (ERPs), number sense, numerical magnitude comparison, mathematical cognition

## Abstract

Although some cognitive studies provided reasons that children with low socioeconomic status (SES) showed poor mathematical achievements, there was no explicit evidence to directly explain the root of lagged performance in children with low SES. Therefore, the present study explored the differences in neural correlates in the process of symbolic magnitude comparison between children with different SESs by the event-related potentials (ERPs). A total of 16 second-graders from low-SES families and 16 from middle/high-SES families participated in this study. According to the results of anterior N1 (early attention) and P2 (extraction of numerical meaning) over the frontal region, the differences among children with different SESs were manifested as differences in general neural activities in terms of attention and top-down cognitive control. In the late stage of cognitive processing, there was no significant difference in the average amplitude of the late positive component (LPC) between children with different SES, indicating that low SES did not influence the information encoding and memory updating of numerical representation, which was responsible by the parietal lobe. The educational implications of this study are mentioned in the discussion.

## Introduction

Although cognitive psychologists and math educators have different definitions of number sense (Gersten et al., 2005), most of them agree that it is an ability to subitize small quantities, discern number patterns, compare numerical magnitudes, estimate quantities, count, and perform simple number transformations (Berch, 2005). Having number sense leads a person from understanding the meaning of numbers to solving complex mathematical problems, from simple numerical comparisons to inventing procedures for performing numerical operations, and from identifying serious numerical errors to using quantitative methods to communicate, process, and explain the information (Berch, 2005). The document “Everybody Counts” of the National Research Council emphasized that the development of number sense is the main aim of mathematics in elementary school.

As a human’s innate ability, number sense has evolutionary significance (Dehaene, 1997). Even babies show a natural sensitivity to small numbers and changes in numbers (Wynn, 1990). Studies confirmed that preschool children already have number sense, such as comprehending basic principles of counting (Gelman and Gallistel, 1986), developing efficient calculation methods spontaneously (Groen and Resnick, 1977), and possessing effective strategies for addition and subtraction (Baroody and Dowker, 2004). After entering elementary school, with the increase of the age and the role of school mathematics education, around the second grade, children’s number sense shows obvious improvement in various ways. Rubinsten et al. (2002) study on children from the beginning of school to the fifth grade found that the size congruity effect did not appear until the end of the first grade (average age, 7.3 years); that is, the consistency of the physical size of two numbers to be compared with their numerical value will affect children’s reaction time (RT) and accuracy (ACC). This result indicated that children began to process symbolic numbers automatically at the end of the first grade. Not only the abovementioned automatic processing of symbolic numbers, but also children’s multi-digit numbers processing at this age has begun to mature. A study using the two-digit comparison task found that, from the second grade (average age, 7.5 years), children’s processing of single digits and two-digit numbers gradually showed an independent and parallel processing mode, which was similar to adults (Nuerk et al., 2004). These results revealed that the representation of multiple digits by second-graders has gradually matured.

The early development of children’s number sense has great impact on their future mathematical abilities and academic achievements (National Mathematics Advisory Panel, 2008). Resorting to a number sense test with five components (i.e., counting, number knowledge, number transformation, estimation, and number patterns), Jordan et al.’s (2006) study found that number sense has a significant predictive effect on children’s subsequent numerical comparison and mathematical problem solving. Children’s scores on number sense in kindergarten are a strong predictor of their mathematics achievements in elementary school (Jordan and Levine, 2009; Jordan et al., 2012). Studies using a magnitude comparison task also confirmed that children’s representation of numerical magnitudes had a significant and positive correlation with their math performance, and the former had a significant predictive effect on the latter (Halberda et al., 2008; Holloway and Ansari, 2009; Mundy and Gilmore, 2009). A study using large-scale online data showed that the effect of number sense on individuals would continue into adulthood (Halberda et al., 2012).

Additionally, children with poorly developed number sense will be exposed to the risk of mathematics learning difficulties (MLDs). Number sense is a powerful predictor of MLDs (Locuniak and Jordan, 2008; Jordan et al., 2010; Seethaler and Fuchs, 2010). Studies found that poor math performance of children with MLDs or developmental dyscalculia (DD) could not be attributed to low intelligence or reading ability (Geary, 2011), because these children had specific deficiencies in basic numerical processing (Passolunghi and Siegel, 2004; Geary et al., 2007; Landerl et al., 2009).

Researchers have conducted a large number of behavioral and event-related potential (ERP) studies to explore the numerical comparison of both adults and children with normal mathematical ability. Moyer and Landauer (1967) were the first to measure the RT in a selection paradigm by pressing the associated button. They found that participants made more mistakes and required more time when the two numbers to be compared were close (e.g., 7 vs. 9) than farther apart (e.g., 2 vs. 9). That is, there is a distance effect (DE) in the process of numerical comparison. The DE also appeared in the classification paradigm in which only one number was included in each trial (e.g., Temple and Posner, 1998; Libertus et al., 2007). By using ERP with high time resolution, several components have been described as being associated with the process of numerical comparison in the classification paradigm. The first negative component, N1, is characterized by a symmetrical distribution in the temporal–parietal sites. This component, which is related to visuospatial attention, has also been associated with numerical comparison (Dehaene, 1996; Gómez-Velázquez et al., 2015). There was no DE in the time window of N1, but the sign effect has shown on this component in the posterior scalp, indicating that N1 represents the stimulus recognition phase of numerical comparison (Dehaene, 1996). The second positive component, P2 (latency of approximately 200 ms after stimulus onset), appears in the frontal and central regions and has been found to be related with task-related processing, such as extraction of numerical meaning (Potts et al., 1996; Potts and Tucker, 2001; Szücs and Csépe, 2004; Chen et al., 2013). The next positive wave, P2p, was initially described by Dehaene (1996) as reflecting the DE (right parietal–occipital–temporal positive waveform to close numbers compared to far numbers) and was seen as an index of the approximate magnitude representation (Hyde and Spelke, 2011). This component, simultaneous with N1, is clearly separated in time and space from the earlier broad fronto-central positivity wave, which is classically known as P2 (Curran et al., 1993). The last positive component is the late positive component (LPC), which is characterized by later onset (usually after 300 ms) and central–parietal distribution (Friedman and Johnson, 2000). Some researchers suggested that this component was the P3b, which indicated the amount of context updating and also reflected the DE (Johnson, 1986; Donchin and Coles, 1988; Grune et al., 1993; Polich, 2007). Moreover, the LPC has also been considered to be related to the process of non-specific motor preparation (Dehaene, 1996; Temple and Posner, 1998; Libertus et al., 2007).

In recent decades, many researchers have been concerned about the variable of “poor family environment” and have conducted a series of studies on children’s cognitive development and promotion under the effect of socioeconomic status (SES). This environmental variable usually includes the economic situation, social resources, social status and rights, and social prestige (American Psychological Association and Task Force on Socioeconomic Status, 2007). In general, children with low SES associated with inadequate educational resources and experiences were often exposed to the dangerous territory of development issues (Bradley and Corwyn, 2002). These children from a disadvantaged environment encountered numerous problems, such as insufficient cognitive development and academic achievement (e.g., White, 1982; Sirin, 2005; Fernald et al., 2011).

Learning opportunities and social experiences linked to SES can influence number sense in early childhood (Jordan and Levine, 2009). A series of longitudinal studies by Jordan et al. (2006, 2007, 2008) revealed that the development of abilities in counting, number relations (recognizing which of two numbers is smaller), and number operations (adding and subtracting small numbers) might be impeded by low income. A study using the 0–10 number line estimation task found that preschool children from low-income families were far less accurate than children with high SES of the same age. Many children with low SES did not even show a correct understanding of the order of numbers (Siegler and Ramani, 2008). For quantitative operation, Griffin et al. (1994) found that 72% of children with high SES could correctly solve the exact arithmetic problems presented with stories, while only 14% of children from low SES backgrounds correctly solved such problems. The difference among children with different SESs was also manifested in the verbal calculation task (Jordan et al., 1992). The researchers believed that this was precisely because children with low SES had weak digital vocabulary knowledge. Therefore, the impact of SES on children’s number sense is mainly reflected in the verbal aspects of mathematics (Dowker, 2005; Jordan and Levine, 2009). Number sense, which is closely related to mathematical achievement, is susceptible to low SES, which can lead to lower math achievement among children from disadvantaged backgrounds (National Mathematics Advisory Panel, 2008).

However, studies on the influence of low SES on children’s number sense were mostly focused on behavioral research using paper-and-pencil number sense test as a tool (Jordan et al., 2006, 2007, 2008). In these previous studies, the exploration of the relationship between low SES and specific components of number sense was mainly focused on number line estimation (e.g., Siegler and Ramani, 2008) and complex calculation (e.g., Griffin et al., 1994). As an important component of number sense, symbolic magnitude comparison is often reflected in children’s daily lives and has been proven by longitudinal study to be a significant predictor of children’s later mathematical achievements (De Smedt et al., 2009). Jordan and Levine (2009) emphasized that as a secondary symbolic number knowledge, numerical magnitude comparison was an important foundation of early mathematics. However, few previous studies have directly examined the performance of children with different SESs on this component of number sense. Therefore, does low SES have the same effect on children’s symbolic numerical comparison? In other words, how does the effect of low SES on the process of numerical comparison occur? In addition, most participants in previous studies were preschool children (e.g., Rubinsten et al., 2002; Nuerk et al., 2004). Does low SES have the same effect on lower grade children who are already very mature in terms of numerical representation? In the exploration of these issues, the ERP technology, which could provide an accurate starting time to different processing stages of numerical comparison, would be used to explore how low SES affects the process of numerical comparison.

Based on the previous ERP studies, three processing stages were recognized in the process of numerical which were the stimulus recognition, the numerical representation and extraction of numerical meaning, and the preparation and implementation of numerical magnitude judgment (Dehaene, 1996). According to the results of previous ERP studies on the selective attention function of the prefrontal cortex in children with low SES (i.e., D’Angiulli et al., 2008; Kishiyama et al., 2008; Stevens et al., 2009), this study proposed the first hypothesis that children with different SES would show variation in the early recognition stage of digit stimuli. In addition, since children with low SES often lag significantly behind in mathematical learning, this study assumed that in the second phase of numerical comparison, children with low SES would show a significant difference from those with middle–high SES. However, previous studies did not find a significant difference on P3b between children with different SES (i.e., Kishiyama et al., 2008), so we believed that in the magnitude comparison task, children with different SES would not show significant difference in this late component, which is dependent on the integrity of the temporal–parietal cortex.

## Materials and Methods

### Participants

Children aged 7–9 years in the second grade were recruited from two primary schools in Yinchuan, China. Initially, 52 children participated in the study. Before EEG data processing, data of 20 children were discarded, among them 11 children failed to complete the experiment (8 of them dropped out of the experiment early due to a cold or physical discomfort, and 3 other children did not complete the experiment due to constant body shaking, talking, and random responses during the experiment), and 9 children’s sufficient trials were less than 70% of the total after the artifact rejection. Finally, data of 32 children were regarded as valid for the analysis: 16 of them were from the low-SES (L-SES) group (8 girls), and the remaining 16 were from the middle/high-SES (M/H-SES) group (8 girls). The mean age was 7.75 years (*SD* = 0.39).

Measures of SES were acquired from children’s parental responses on the Socioeconomic Status Questionnaire, which was based on the programme for international student assessment (PISA) in 2018 (Organization for Economic Cooperation and Development, 2018) and was referred to the studies of Fang et al. (2008) and Fan et al. (2012). The SES criteria consisted of parental education, occupation, and family resources, which together represented SES better than any of these alone (White, 1982). The parents of children in the M/H-SES group obtained, on average, undergraduate education, whereas those in the L-SES group only reached the primary level (years of education: *M*_*M/H–SES*_ ± *SD* = 17.79 ± 2.39, *M*_*L–SES*_ ± *SD* = 7.14 ± 1.83). There was a significant difference in the years of education between the two groups [*t*(26) = 13.21, *p* < 0.001]. Parental occupation was measured by using the International Socio-Economic Index of Occupational Status (Ganzeboom and Treiman, 1996). Family resources included a computer, television, refrigerator, telephone/cellphone, washing machine, bathroom, bath facilities, kitchen, heating installation, and 11 other resources, which were basic and necessary for family life and greatly important to children’s studies. The calculation steps for SES were as follows (see Fan et al., 2012): (i) assigning values to the variables including parents’ education, occupation, and family resources; (ii) transforming the abovementioned assigned variables, that is, the one who has higher education or higher class occupation scores of the parents is selected as the representative for parents’ education or occupation; (iii) utilizing the Item Response Theory to perform parameter estimation on family resources, in which the parameter estimation index is acquired; (iv) dealing with the missing values of variables with the following method: if a participant has more than two missing variables, then he or she will be regarded as a missing sample; if a participant only has one missing variable, then we will compute the corresponding values to substitute the missing value by regression analysis with two other variables; and (v) transforming education, occupation, and family resource into standard scores. Then, a main component analysis was conducted, and the following formula was used to obtain the SES score: $\text{SES}=\frac{\beta_{1}\,\times \,Z_{e\,d\,u\,c\,a\,t\,i\,o\,n}+\beta_{2}\,\times Z_{o\,c\,c\,u\,p\,a\,t\,i\,o\,n}+\beta_{3}\,\times \,Z_{f\,a\,m\,i\,l\,y\,r\,e\,s\,o\,u\,r\,c\,e}}{\varepsilon_{\text{f}}\,}$. β_1_, β_2_, and β_3_ are the factor loadings. ε_*f*_ is the root of the eigenvalues of the first factor. The participants came from Yinchuan, an inland city in the northwest region of China, which had a relatively backward level of economic development. Thus in this study, a high score on the SES questionnaire only represented a middle to high level of SES.

The participants in the current study had normal general cognitive ability, tested by Raven’s Standard Progressive Matrices (R’SPM-CR), revised by Zhang and Wang (1989), and had normal verbal intelligence, tested by the verbal subscale of the McCarthy Scales of Children’s Abilities (MSCA-CR), revised by Chen and Li (1994). There was no significant difference in the scores in general cognitive ability (R’SPM-CR: L-SES *M* = 37.00, *SD* = 5.92; M/H-SES *M* = 40.63, *SD* = 4.46, *t*(30) = −1.96, *p* > 0.05, 95% *CI* = [−7.41, 0.16]) and verbal intelligence (MSCA-CR: L-SES *M* = 77.06, *SD* = 9.77; M/H-SES *M* = 80.56, *SD* = 5.61, *t*(30) = −1.24, *p* > 0.05, 95% *CI* = [−9.25, 2.25]) between the two SES groups. More detailed information of the participants was shown in Table 1. The participants, as assessed by parental report, had no history of neurological or psychiatric disorders or perinatal substance abuse. This study obtained the written informed consent of each child’s guardian and was approved by the Academic Ethics Committee of the School of Psychology, Shaanxi Normal University, China.

**TABLE 1:** The general cognitive abilities and the demographic characteristics of the participants in different SES groups (*M* ± *SD*).

	***n*(female)**	**Mean age**	**R’SPM**	**MSCA (verbal)**	**SES**	**Parental education^*a*^**
Range	/	7.42–9.08	28.00–50.00	58.00–89.00	−2.05–2.46	5–21
L-SES	16(8)	7.92 ± 0.47	37.00 ± 5.92	77.06 ± 9.77	−1.43 ± 0.23	7.44 ± 1.63
H/M-SES	16(8)	7.99 ± 0.29	40.63 ± 4.46	80.56 ± 5.61	1.62 ± 0.84	17.13 ± 3.54

*SES, socioeconomic status; L-SES, low SES; M/H-SES, middle/high SES, R’SPM, Raven’s Standard Progressive Matrices; MSCA, McCarthy Scales of Children’s Abilities. ^*a*^Parental education refers to the parent who has higher education*.

### Stimuli and Task

According to the selection paradigm (Moyer and Landauer, 1967), the magnitude comparison task was presented to each participant with two single digits displayed simultaneously during the recording session. The stimuli of this task consisted of 10 pairs of Arabic numerals from 1 to 9 (except number “5”), with no repeating digits in each pair (e.g., “3 3”). They were displayed in white Arial font in the center of the computer screen with black background. There were two different numerical distances: 5 (i.e., 1–6, 2–7, 3–8, 4–9) and 1 (i.e., 1–2, 2–3, 3–4, 6–7, 7–8, 8–9). The position where the two numbers appeared was counterbalanced, half on the left and half on the right. There were three blocks of 48 stimuli (144 trials in total; 72 trials per distance condition). The participants were required to decide which of the two numbers was larger by pressing the button on the corresponding side as quickly and accurately as possible. The participants were seated 70 cm away from the computer screen. The horizontal visual angle of the stimuli was 2.01°.

### Procedure

**FIGURE 1:**
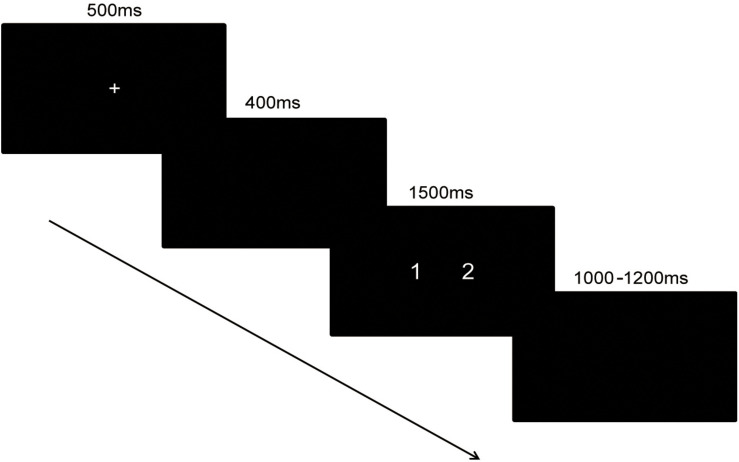
The time course of magnitude comparison task.

In the magnitude comparison task, the time interval between the two blocks was 1 min. Each trial began with a fixation sign shown for 500 ms. After a 400 ms pause, a pair of numbers was shown for 1,500 ms. Then, a black screen with a variable interval ranging from 1,000 to 1,200 ms appeared. The trials in the block were presented in pseudo-random order. The same response consecutively occurred no more than two times. The time assignment of the task was shown in Figure 1.

Participants were tested individually. After preparation for the ERP experiment, such as cleaning and blow-drying the hair, EEG/EOG sensor electrodes were attached, and the experimenter informed the participant of the experimental instruction in detail. The participant was seated 70 cm away from the computer screen. The experiment began with 10 practice trials, which were not included in the statistical analysis, to familiarize participants with the experimental requirements. There were no formal experimental number pairs in the practice trials. The experiment lasting approximately 1 h for each participant, including electrode placement, practice, and ERP recording sessions. In the present study, E-Prime 2.0 software (Psychology Software Tools Inc., Sharpsburg, PA, United States) was used to present the stimuli and collect behavioral data.

### ERP Recording and Data Analysis

The ERP was recorded and analyzed with SCAN 4.5 software (Neuro Scan, Inc., Herndon, VA, United States) from a 64-channel Ag/AgCl electrode Quick-Cap that was extended according to the 10–20 system. EEG was physically referenced to the left mastoid and then was re-referenced offline to the average of the left and right mastoid. An equidistant point between FPZ and FZ served as the location of the ground electrode. EOG was recorded with electrodes located at points 2 cm outside both canthi and above and below the left eye to monitor the horizontal and vertical eye movements, respectively. EEG and EOG were continuously digitized at a sampling rate of 500 Hz using the SynAmps2 amplifier (Neuro Scan, Inc.) with a band pass of 0.05–100 Hz and stored in a hard disk for offline analysis. Electrode impedance was maintained below 5 KΩ.

Two ERP averages were calculated for each participant: one for the trials with small number distance and another for the trials with large number distance. Epochs extended from 100 ms pre-stimulus to 800 ms post-stimulus. The baseline correction was related to the averaged interval between −100 and 0 ms. A band-pass filter was set from 0.05 to 30 Hz (zero-phase, 24 dB/octave). Trials with artifacts (voltage exceeding ±100 μV in any channel) that were caused by eye blinks, eye movement, or muscle potentials and trials with incorrect behavioral responses were excluded from the ERP averages, resulting in an exclusion of approximate 17.71% of the trials from the average. The proportion of trials in the analysis ranged from 68.06 to 90.28%. Finally, the average number of trials that were included in the analysis in each condition was 60.98 for large distance and 57.52 for small distance. The data were processed with Neuroscan Edit program and Origin Pro 8 (OriginLab, Inc., United States).

Time windows and the electrodes for analysis were selected based on previous studies and observation results of the data. After observation of the grand average waveform, it was found that the first component evoked by the selection paradigm in this study was N1, occurring in the frontal and central regions. This anterior N1, separated from the posterior N1, is regulated by top-down control of the prefrontal cortex and reflects the degree of voluntary attention (Luck, 1995; Barceló et al., 2000; Yago et al., 2004). In the previous study, the anterior N1 was also evoked in the process of mental arithmetic (e.g., Kong et al., 1999; Yagoubi et al., 2003). Finally, to explore the time course of magnitude comparison of the children with different SESs, three components were selected: the anterior N1 (100–140 ms, F3/FZ/F4/FC3/FCZ/FC4/C3/CZ/C4) (Kong et al., 1999; Yagoubi et al., 2003; Kishiyama et al., 2008), the P2 component (200–250 ms, F3/FZ/F4/FC3/FCZ/FC4/C3/CZ/C4) (Szücs and Csépe, 2004; Chen et al., 2013), and the LPC (450–700 ms, F3/FZ/F4, C3/CZ/C4, P3/PZ/P4) (Grune et al., 1993; Turconi et al., 2004; Libertus et al., 2007; Han et al., 2017). The number of electrodes selected for the analysis was the same in different groups.

### Statistical Analysis

For the behavioral data (RT and ACC), to explore the differences in DE between the two SES groups, an SES (L-SES vs. M/H-SES) × numerical distance (large vs. small) repeated-measures ANOVA was conducted. SES was a between-subject variable, and numerical distance was a within-subject variable.

For the ERP data, in order to explore the differences in the neural mechanism between the L-SES and M/H-SES groups, the average amplitude over the analysis electrodes in each time window was calculated, respectively. For the average amplitude of the N1 and P2, an SES (L-SES, M/H-SES) × numerical distance (large, small) × location (frontal, fronto-central, and central) repeated-measurement ANOVA was performed, respectively. For the average amplitude of the LPC, an SES (L-SES, M/H-SES) × numerical distance (large, small) × location (frontal, central, and parietal) repeated-measurement ANOVA was conducted. The numerical distance and location were the within-subject variables. To investigate the changes in the ERP components in different brain regions, the amplitudes of the electrodes in different hemispheres of the same location were averaged together (e.g., F3/FZ/F4). In the present study, Greenhouse–Geisser correction was performed in all behavioral and physiological ANOVAs as necessary. Original *F* and *df*, $\eta_{\text{p}}^{2}$ of effect size index, and corrected *p*-values are reported in the results when necessary.

## Results

### Reliability Analysis

The current study conducted a correlation analysis to explore the test–retest reliabilities in RT and ACC between the first- and second-half trials of the experimental task. The results showed that the task was extremely reliable for both RT (*r* = 0.97, *p* < 0.001) and ACC (*r* = 0.81, *p* < 0.001), suggesting good task reliability (Maloney et al., 2010; Miller and Ulrich, 2013).

### Behavioral Results

Reaction time that exceeded three standard deviations in each condition was eliminated. Both RT (ms) and ACC (%) were entered into the SES (2) × numerical distance (2) repeated-measurement ANOVA. The results were presented in Table 2.

**TABLE 2:** The average reaction time (RT, ms) and accuracy (ACC, %) in different conditions of children with different SESs.

	**Large distance**		**Small distance**	
	**RT [95% *CI*] (*SD*)**	**ACC [95% *CI*] (*SD*)**	**RT [95% *CI*] (*SD*)**	**ACC [95% *CI*] (*SD*)**
L-SES	669 [605, 732] (132)	96.70 [95, 99] (3.47)	754 [686, 822] (143)	89.67 [86, 94] (9.02)
M/H-SES	646 [558, 709] (115)	96.88 [95, 99] (4.10)	726 [658, 794] (122)	91.41 [88, 95] (5.81)

#### Reaction Time

A significant main effect of numerical distance was found [*F*(1,30) = 120.95, *p* < 0.001, $\eta_{\text{p}}^{2}$ = 0.801], suggesting that RT for the small distance was significantly longer than that for the large distance. The SES main effect [*F*(1,30) = 0.32, *p* > 0.05, $\eta_{\text{p}}^{2}$ = 0.011] and interaction of SES × numerical distance [*F*(1,30) = 0.12, *p* > 0.05, $\eta_{\text{p}}^{2}$ = 0.004] were not significant.

#### Accuracy

The main effect of numerical distance was significant [*F*(1,30) = 34.11, *p* < 0.001, $\eta_{\text{p}}^{2}$ = 0.532]. The ACC of judging the numbers with a small distance was significantly lower than that with a large distance. Both the SES main effect [*F*(1,30) = 0.27, *p* > 0.05, $\eta_{\text{p}}^{2}$ = 0.009] and SES × numerical distance interaction [*F*(1,30) = 0.53, *p* > 0.05, $\eta_{\text{p}}^{2}$ = 0.017] were not significant.

The behavioral results suggested that the DE existed in both groups; that is, children were faster and more accurate when judging numbers with large distance compared with the small distance. However, there was no significant difference in the behavioral indices of the magnitude comparison task between children with different SESs.

### ERP Results

**FIGURE 2:**
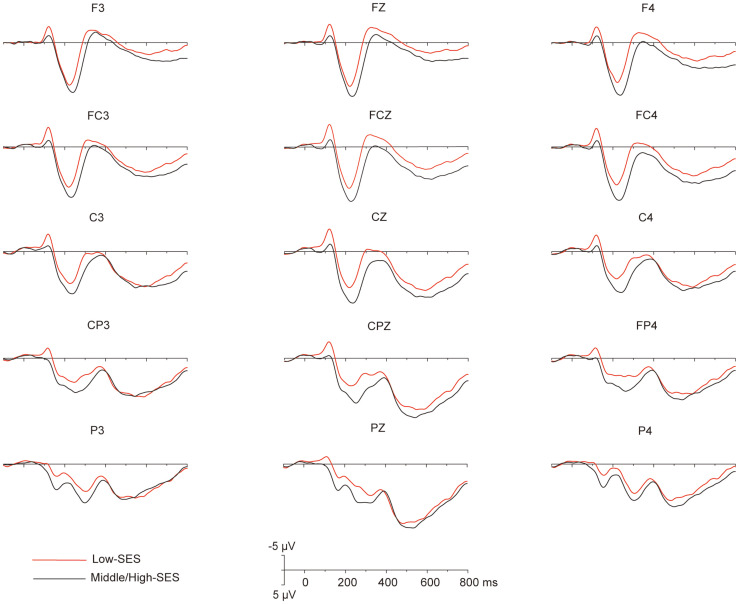
The grand average (*n* = 32) for the different SES groups in each electrode that to be analyzed.

Grand average ERPs over the 32 participants elicited by the magnitude comparison task showed a sequence of events. Three major components were identified after stimulus onset: a first negative component over frontal to central sites, the N1, peaking around 120 ms; an anterior positivity, the P2, peaking around 220 ms and maximal over frontal electrodes; and a late positivity over frontal–central–parietal sites, LPC, peaking around 570 ms (see Figure 2). Overall, the dissociation of the ERP amplitude between children with L-SES and H/M-SES appeared around 100 ms after stimulus presentation, and the differences in the amplitudes between the two SES groups were distributed in different time windows.

#### 100–140 ms Time Window

**FIGURE 3:**
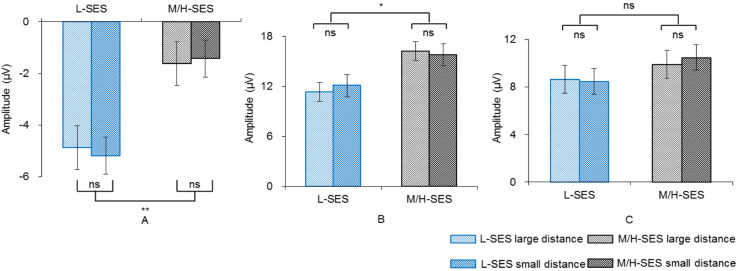
The average amplitudes of the children with different SES in different conditions for three time windows. **(A)** 100–140 ms, **(B)** 200–250 ms, **(C)** 450–700 ms; ns, no significance, *p < 0.05, **p < 0.01. L-SES, low SES; M/H-SES, middle/high SES.

**FIGURE 4:**
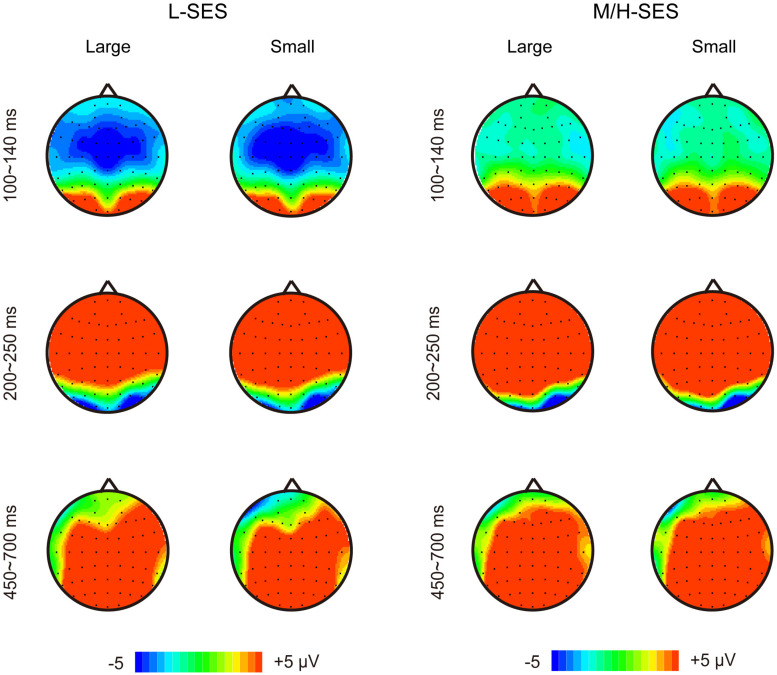
The topographical maps for the children with different SES in different conditions. L-SES, low SES; M/H-SES, middle/high SES; Large, large distance; Small, small distance.

In the 100–140 ms time window, an SES (2) × numerical distance (2) × location (3) repeated-measurement ANOVA was conducted. The results showed that the SES main effect was significant, revealing that children with L-SES showed more negative amplitude than the children with M/H-SES [*F*(1,30) = 12.85, *p* < 0.01, $\eta_{\text{p}}^{2}$ = 0.300] (see Figures 3A, 4). The main effects on both numerical distance and location were not significant [distance: *F*(1,30) = 0.01, $\eta_{\text{p}}^{2}$ = 0.001; location: *F*(2,60) = 0.79, $\eta_{\text{p}}^{2}$ = 0.026, *p*s > 0.05] (see Figure 3A and Table 3). The interactions were not significant (*p*s > 0.05).

**TABLE 3:** The average amplitudes of different locations in each time window during the numerical comparison (μV) (*M* ± *SD* [95% *CI*]).

**Locations**	**100–140 ms**	**200–250 ms**	**Locations**	**450–700 ms**
Frontal	−3.08 ± 0.50 [−4.10, −2.06]	14.74 ± 0.81 [13.08, 16.39]	Frontal	4.32 ± 0.74 [2.82, 5.83]
Fronto-central	−3.45 ± 0.54 [−4.57, −2.34]	14.66 ± 0.90 [12.83, 16.49]	Central	11.23 ± 0.89 [9.41, 13.05]
Central	−3.30 ± 0.51 [−4.35, −2.25]	12.25 ± 0.90 [10.41, 14.08]	Parietal	12.56 ± 1.01 [10.50, 14.62]

#### 200–250 ms Time Window

In the 200–250 ms time window, the results of the SES (2) × numerical distance (2) × location (3) repeated-measurement ANOVA revealed that the SES main effect was significant, suggesting that the children with M/H-SES showed more positive amplitude than their counterparts [*F*(1,30) = 6.55, *p* < 0.05, $\eta_{\text{p}}^{2}$ = 0.179]. The main effect of numerical distance was not significant [*F*(1,30) = 0.09, *p* > 0.05, $\eta_{\text{p}}^{2}$ = 0.003] (see Figures 3B, 4). The differences of average amplitudes among different locations were significant [*F*(2,60) = 25.52, *p* < 0.001, $\eta_{\text{p}}^{2}$ = 0.460]. A *post hoc* test revealed that the amplitudes in frontal and fronto-central regions were significantly larger than that in the central region (*p*s < 0.001) (see Table 3). The interactions were not significant (*p*s > 0.05).

#### 450–700 ms Time Window

In the 450–700 ms time window, an SES (2) × numerical distance (2) × location (3) repeated-measurement ANOVA was performed. The results indicated that the SES main effect was not significant [*F*(1,30) = 1.18, *p* > 0.05, $\eta_{\text{p}}^{2}$ = 0.038]. The main effect of numerical distance was not significant [*F*(1,30) = 0.18, *p* > 0.05, $\eta_{\text{p}}^{2}$ = 0.006] (see Figures 3C, 4). The main effect of location was significant [*F*(2,60) = 61.35, *p* < 0.001, $\eta_{\text{p}}^{2}$ = 0.672]. A *post hoc* test showed that the average amplitudes increased from the frontal to the parietal lobe, and the amplitudes in the central and parietal regions were significantly larger than that in frontal region (*p*s < 0.001) (see Table 3). The other interactions were not significant (*p*s > 0.05).

## Discussion

A series of studies on the relationship between mathematical ability and SES have shown that, compared with children with middle/high SES, children with low SES were disadvantaged and were more likely to be at high risk in mathematics learning. The results of the present study provided evidence that there were neural correlate differences between children with different SESs. In the following, we discuss the particular analyses and neurocognitive indices supporting this claim.

Based on the behavioral data of RT and ACC, second-grade children showed a numerical DE where they judged the numbers with large distance much faster and more accurately, which was consistent with previous studies (e.g., Soltész et al., 2007; Gebuis et al., 2009). These findings indicate that there exists a “mental number line” for children in the process of numerical comparison. Children might judge the magnitudes according to the to-be-compared number’s position on this mental number line. When the to-be-compared numbers were close to each other, more greater overlapping areas of these two numbers on the mental number line would make it more difficult to distinguish one from the other. Conversely, it was much easier to compare two numbers when they were more distant from each other. Nevertheless, children in different SES groups showed extremely minor differences in RT and ACC on the magnitude comparison task (RT: 685.63 ms for M/H-SES, 711.16 ms for L-SES; ACC: 95.15% for M/H-SES, 93.19% for L-SES). One reason is that the experimental task is relatively easy for second-graders (in the second semester), so the “ceiling effect” appears.

In the ERP with the advantages of high time resolution, children with different SESs showed significant differences in EEG activity at different stages when performing the magnitude comparison task. In this study, the N1 component appeared in the frontal and central regions. According to the time and brain regions of occurrence, this component was the anterior N1. This result was inconsistent with a previous study (i.e., Dehaene, 1996) in which the posterior N1 was evoked by the number comparison task. Researchers held that the anterior N1, which has the same generators as the P1 component, originates from the ventral dorsolateral extrastriate cortex (Clark et al., 1994; Heinze et al., 1994) and is regulated by top-down control of the prefrontal cortex (Barceló et al., 2000; Yago et al., 2004). The reasons that the N1 component is evoked in different brain regions are because of the differences in the form of the stimulus presentation and in the comparison baseline between different experiments. In previous studies (e.g., Dehaene, 1996; Gómez-Velázquez et al., 2015), the classification paradigm was used to explore the process of numerical comparison. In this task, only one digit was presented in each trial, without changing the baseline number to be compared (e.g., “5”). In the present study, each trial of the task included two digits simultaneously (e.g., “2” and “7”). As a result, participants firstly needed to accurately represent each number on their mental number line, instead of making classification judgments based on a reference point. The difficulty of this task has increased to some extent, therefore, the involvement of prefrontal cognitive control is required (Miura et al., 2016; Siciliano et al., 2017). Moreover, in the ERP studies exploring the process of mental arithmetic, two numbers presented simultaneously in each trial, anterior N1 was evoked (e.g., Kong et al., 1999; Yagoubi et al., 2003). Through evaluating the prefrontal function of children with different SESs by observing the anterior N1 amplitude for the visual stimuli, Kishiyama et al. (2008) has found that children with low SES have more insufficient ability to allocate attention resources and inhibit irrelevant stimulus interference. The present study confirmed the conclusion that children with low SES showed more negative amplitude between 100 and 140 ms after the digits occurred, indicating that when these children perceived and recognized the numbers, they needed to invest more attention resources to suppress the interference of irrelevant information to their intentional attention to catch up with children with middle/high SES. Therefore, the current study suggests that low SES may be one of the important variables affecting children’s attention assignment to the digit stimuli.

Another finding was that the average amplitude of children with low SES in the 200–250 ms time window was significantly smaller than that of children with middle/high SES. Previous studies found that the P2 component, which appeared in the frontal–central regions after the N1 component, was related to the early recognition of the target, reflecting the processing related to the task (Potts and Tucker, 2001; Potts et al., 1996). The latency of the P2 component is approximate 200–300 ms, which is related to the early semantic processing of visual information (Zhao, 2010). In this study, the average amplitude of the P2 was maximal over frontal electrodes. According to previous studies, the P2 concentrated in the frontal–central regions mainly reflects the process of perception analysis, such as rapid extraction of numerical meaning in a number-related task, while the enhancement in the amplitude of the P2 may be related to experience and practice (Szücs and Csépe, 2004; Amodio, 2010). The difference in P2 amplitude between children with different SES suggested that children from different SES families might be different in the recognition of target numbers and early semantic processing of abstract Arabic numerals.

Human’s prefrontal cortex has a long period of postnatal development (e.g., Casey et al., 2000; Fuster, 2002), so the external environment, such as environmental deprivation and stress (Braun et al., 2000) and environmental complexity (van Praag et al., 2000), will affect the development of the prefrontal cortex. Various factors associated with L-SES rearing conditions may influence the brain development of children. First of all, compared with M-SES children, L-SES children often live in cognitively impoverished environments, which leads them to a lack of cognition stimulating materials and experiences, and receive less attention from adults (Bradley and Corwyn, 2002). In addition, these children from L-SES families often experience higher levels of stress. They are exposed to more chronic stressors in the family, such as prolonged poverty and parental strife, and therefore have higher basal levels of the stress hormone corticosteroids and poorer selective attention (Lupien et al., 2001).

In the present study, an ERP difference among children with different SESs did not appear in the average amplitude of LPC (in the 450–700 ms time window), which was consistent with a previous study (i.e., Kishiyama et al., 2008). The LPC, which is characterized by later onset (usually after 300 ms) and central–parietal distribution (Friedman and Johnson, 2000), represents the amount of context updating and is found to be sensitive to the arithmetic relatedness (e.g., Johnson, 1986; Donchin and Coles, 1988; Polich, 2007; Galfano et al., 2011). The results revealed that low SES, as an external environmental variable, did not exert an influence on the stage of children’s information encoding and memory storage associated with numerical representation. Moreover, the amplitude of LPC increasing from the frontal to the parietal lobe, indicated that the parietal region was more involved in the more complex deep processing related to mathematics. Although fMRI studies found that children with MLD had impairment in the bilateral intraparietal sulcus (IPS) involved in number magnitude processing (Habib, 2000; Mussolin et al., 2014), and had significant differences in the P300 latency and amplitude compared to normal children (Soltész et al., 2007; Taylor and Keenan, 2010; Wang et al., 2015), in this study, children with L-SES were not MLD, and some of them even had higher math scores in the class. Therefore, as a distal factor, SES is not enough to affect the deep numerical processing in the parietal lobe. However, in the present study, children with different SESs did not show a numerical DE on the average amplitude in this time window, which may be due to the differences in the experimental tasks. For this reason, it is necessary to compare the ERP results with different forms of magnitude comparison tasks in the future.

To sum up, the time course of numerical processing can be combined with corresponding brain regions to explain the discrepancies among children with different SES. Previous studies have found that prefrontal cortex activation comes first compared with parietal cortex during arithmetic processing (Menon et al., 2000; Brass et al., 2005). The anterior component is related to the cognitive control processing of the prefrontal cortex, while the parietal component reflects the involvement of numerical representation (Dehaene, 1996; Pinel et al., 2001). Therefore, according to the results of the N1 (early attention) and the P2 (extraction of numerical meaning) over the frontal region, the differences among children with different SESs were manifested as differences in general neural activities in terms of attention and top-down cognitive control. The findings in this study provide some valuable suggestions to researchers and educators about the mathematical education of children with low SES. It is insufficient and unscientific to only provide children with low SES with books and toys. Teachers and tutors should pay more attention to the fundamental cognitive functions of these children, such as adding training contents of intentional attention about the numbers in the curriculum for these children.

## Conclusion

In the current study, according to the results of the anterior N1 (early attention) and P2 (extraction of numerical meaning) over the frontal region, the differences among children with different SESs were manifested as differences in general neural activities in terms of attention and top-down cognitive control. When children with low SES perceived and recognized numbers, they needed to invest more attention resources to suppress the interference of irrelevant information to their intentional attention, so as to catch up with the children with middle/high SES. Moreover, these children from low-SES families showed significant differences in the semantic processing and meaning extraction of abstract Arabic numerals compared to the children with middle/high SES. In the late stage of cognitive processing (450–700 ms), there was no significant difference in the average amplitude of the LPC among children with different SES, indicating that low SES did not influence information encoding and memory updating of numerical representation, which was responsible by the parietal lobe.

## Data Availability Statement

The datasets generated for this study are available on request to the corresponding author.

## Ethics Statement

The studies involving human participants were reviewed and approved by the Academic Ethics Committee of the School of Psychology, Shaanxi normal University, China. Written informed consent to participate in this study was provided by the participants’ legal guardian.

## Author Contributions

QB and LZ were responsible for the formulation of the research program. In addition, QB was responsible for data collection, processing, and manuscript writing. LZ was responsible for the manuscript review. YL as the third author of this study was responsible for the revision of this manuscript. YZ and GS assisted in the conduct of the experiment. All authors contributed to the article and approved the submitted version.

## Conflict of Interest

The authors declare that the research was conducted in the absence of any commercial or financial relationships that could be construed as a potential conflict of interest.
